# The intake–adequacy gap in dysphagia nutrition: texture-modified foods, nutrient delivery and realised intake in older adults

**DOI:** 10.3389/fnut.2026.1897745

**Published:** 2026-08-17

**Authors:** Umar Shah, Darren Greetham, Steffen Hirth, Sadia Javed, Yashaswini Premjit, Pattanapong Tiwasing, John Lubaale, Haroon Rashid Naik, James Ball, Preston Walker, Rewati Raman Bhattarai, Stuart K. Johnson

**Affiliations:** 1 School of Food Science and Nutrition, Faculty of Environment, University of Leeds, Leeds, United Kingdom; 2 School of Earth, Environment and Sustainability, Faculty of Environment, University of Leeds, Leeds, United Kingdom; 3 Nottingham University Business School, University of Nottingham, Nottingham, United Kingdom; 4 Department of Health and Nutrition, School of Health, Life Science and Education, University College Birmingham, Birmingham, United Kingdom; 5 Division of Food Science and Technology, Sher-E-Kashmir University of Agricultural Science and Technology, Srinagar, Kashmir, India; 6 Oak House Kitchen, Birmingham, United Kingdom; 7 School of Molecular and Life Sciences, Faculty of Science and Engineering, Curtin University, Perth, WA, Australia; 8 Ingredients by Design Pty Ltd, Perth, WA, Australia

**Keywords:** care homes, dysphagia, intake–adequacy gap, malnutrition, texture-modified foods

## Abstract

Dysphagia is common among older adults in long-term care and is frequently managed using texture-modified foods (TMFs) aligned with the International Dysphagia Diet Standardisation Initiative (IDDSI). Although texture modification improves swallowing safety and standardisation, nutritional adequacy is often inferred from planned provision rather than verified at the level of actual consumption. This distinction is clinically important because swallowing impairment, fatigue, prolonged mealtimes, reduced appetite, sensory decline and dependence on feeding assistance limit the amount of food consumed. As a result, TMFs may meet prescribed texture requirements and planned nutritional targets while failing to deliver sufficient energy, protein and key micronutrients, including calcium, vitamin D, vitamin B12, iron and zinc, in practice. This review examines this mismatch as an intake–adequacy gap in dysphagia nutrition. We synthesise evidence on reduced intake, plate waste, nutrient-density loss during texture modification, sensory acceptability and limitations in care-home monitoring. We argue that nutritional adequacy in dysphagia care should be evaluated as an intake-realised outcome shaped by food composition, swallowing safety, sensory engagement and mealtime support. Reframing TMFs as structured nutritional interventions may help align texture compliance with nutrient delivery, identify where planned nutrition is lost before consumption, and guide targeted strategies to improve nutritional outcomes among older adults with dysphagia.

## Highlights

- Texture compliance alone is insufficient to establish nutritional adequacy in dysphagia care.
- The intake–adequacy gap explains how planned nutritional provision may diverge from realised intake.
- Future texture-modified foods should be designed to optimise nutrient density, acceptability and intake-realised nutritional delivery.

## Introduction

Dysphagia is highly prevalent among older adults and is particularly common in residential care homes, where frailty, neurodegenerative disease, stroke and functional decline frequently converge (1, 2). Texture-modified foods (TMFs) and thickened fluids, therefore, form the central component of dysphagia management because they reduce the risk of aspiration and choking while supporting oral intake (3). Over the last decade, the global adoption of the International Dysphagia Diet Standardisation Initiative (IDDSI) has substantially improved the safety, consistency and communication of dysphagia diets through a standardised framework for food texture and fluid thickness (4, 5). Consequently, swallowing safety is now more systematically operationalised across clinical and long-term care settings (6, 7).

Nutritional care is similarly guided by established dietary frameworks, and menu planning is intended to maintain adequate energy, protein, and micronutrient provision. In the UK, these include Dietary Reference Values (DRVs), encompassing Estimated Average Requirements (EARs), Reference Nutrient Intakes (RNIs), and food-based guidance such as Eatwell (8–13). However, although these guidelines define population-level dietary requirements, they are primarily structured around planned provision rather than realised intake. This distinction is particularly important in dysphagia care because eating capacity is frequently constrained by swallowing impairment, fatigue, prolonged mealtimes, reduced sensory tolerance, and dependence on feeding assistance (14, 15).

Despite advancements in dysphagia management, malnutrition remains common among older adults consuming TMFs. Wu et al. (16) reported significantly lower energy intake among individuals consuming TMFs compared with those consuming regular foods, with a pooled mean difference of approximately 238 kcal/day. The study further identified reduced protein intake, increased plate waste, prolonged mealtimes, and inadequate intake of several micronutrients among TMF consumers. Shimizu et al. (17) reported a significantly higher prevalence of poor appetite among older adults consuming TMFs in post-acute rehabilitation, with TMF consumption independently associated with poor appetite. These findings are clinically important because dysphagia frequently limits the ability to compensate for reduced intake through larger meal volumes. Nutritional adequacy is therefore determined not only by what is theoretically provided, but by the nutrient density and acceptability of food actually consumed. This distinction highlights the broader conceptual shift from provision-based interpretations of adequacy towards intake-realised models of nutritional assessment in dysphagia care (Table 1).

**Table 1:** Reframing nutritional adequacy in dysphagia care: From provision-based to intake-realised perspective.

Conceptual dimension	Provision-based interpretation	Intake-realised interpretation
Definition of adequacy	Nutritional adequacy inferred from planned provision aligned with dietary reference standards	Nutritional adequacy is determined by nutrients actually consumed under conditions of constrained intake
Unit of evaluation	Menu composition and theoretical daily provision	Realised nutrient exposure at the level of an eating episode
Basis of assessment	Documentation, menu design and nutritional screening frameworks	Observed intake, acceptability and realised intake patterns
Function of TMFs	Vehicles for delivering prescribed nutrients within texture restrictions	Structured systems in which composition and physical organisation jointly shape intake and nutrient delivery
Role of texture compliance	Primary indicator of safety and quality assurance	Necessary condition for safe swallowing, but insufficient to ensure nutritional adequacy
Detection of nutritional risk	Identified retrospectively through weight change or screening scores	Emerges prospectively through reduced intake, meal completion and consumption behaviour
Interpretation of outcomes	Adequacy is assumed where the provision meets nutritional targets	Adequacy contingent on the alignment between provision, intake and realised exposure
System orientation	Emphasis on provision, compliance and auditability	Emphasis on the interaction between food systems, individual behaviour and nutritional outcomes
Operational measurement	Nutrient content estimated from menus, recipes or planned portions	Nutrient exposure is estimated by combining the nutrient composition of served food with meal completion, plate waste and actual consumption

Similarly, the routine food preparation processes further weaken the relationship between nutritional provision and intake. For example, to achieve an IDDSI-compliant texture level, TMFs are commonly blended, diluted, sieved, and stabilised by starch- or gum-based thickening agents. However, dilution during texture modification reduces nutrient density per mouthful, particularly when water, stock or gravy is added during preparation (16). Reheating and cook–chill systems can additionally reduce heat-sensitive micronutrients such as vitamin C and folate, whereas homogenisation can diminish visual appeal and flavour contrast, reducing appetite and meal satisfaction among older adults consuming TMFs (18–20). Such factors further compromise dietary intake under conditions in which intake capacity is already limited.

Therefore, foods that are safe to swallow may nevertheless be insufficient to provide adequate nutrient intake in practice. TMFs may comply with prescribed texture requirements, yet still fail to provide adequate nutritional exposure at the point of consumption. This mismatch is represented as an intake–adequacy gap, arising not from deficiencies in swallowing safety, but from the challenge of translating planned dietary provision into sufficient realised intake within routine dysphagia care.

**Figure 1:**
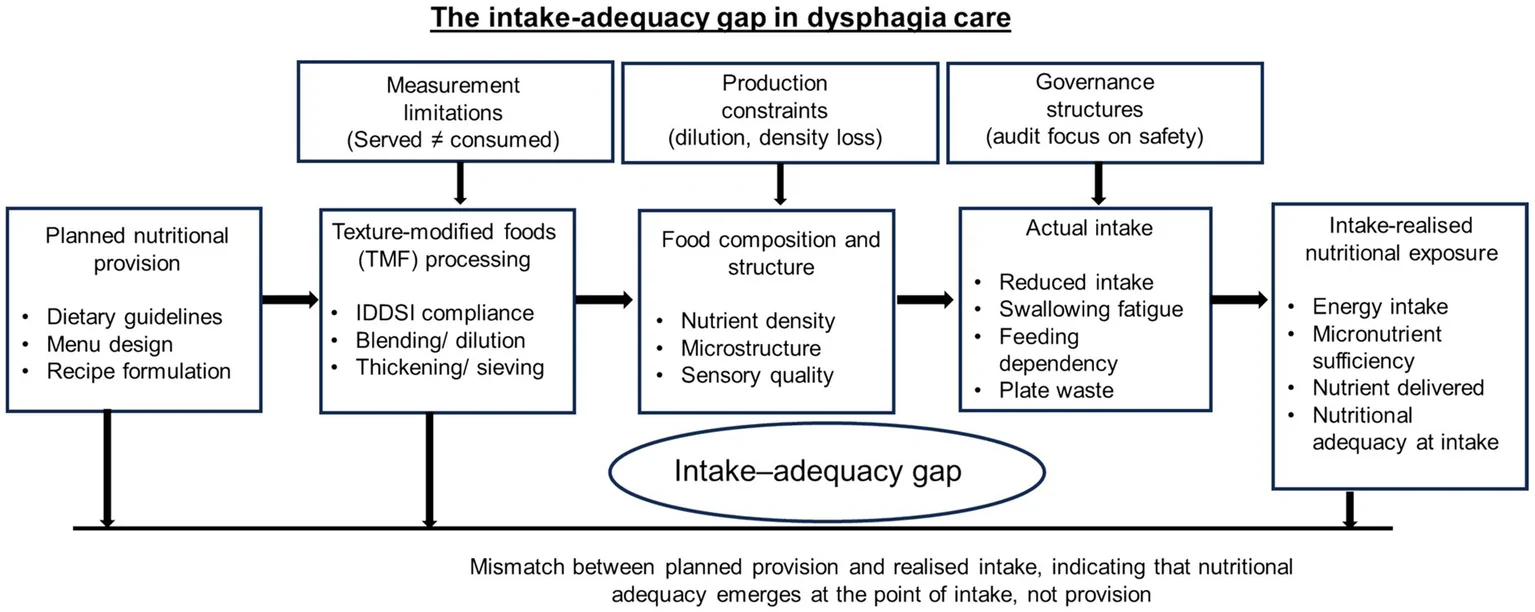
Conceptual framework of the intake–adequacy gap in dysphagia care. Schematic illustrating mismatch between planned nutritional provision and intake-realised nutritional exposure, arising from interacting limitations in measurements, production, and governance.

In this review, the intake–adequacy gap refers to the difference between the nutritional value of planned texture-modified foods (TMFs) and the nutrients actually consumed during routine meals. It may be assessed using measures such as meal completion, plate waste, food refusal, feeding assistance, acceptability, body weight and nutritional screening outcomes. Research settings may additionally use weighed food records, nutrient analysis, direct observation and relevant biochemical indicators. This gap links planned nutritional provision with realised nutrient intake and may contribute to malnutrition, frailty and functional decline among older adults with dysphagia. The review therefore examines how limitations in measurement, food production and governance influence the intake–adequacy gap and argues that nutritional adequacy should be evaluated as an intake-realised outcome rather than solely as a property of food provision. These relationships are summarised in Figure 1.

## Methods

This review adopts a narrative review approach to synthesise evidence from food science, nutrition, clinical practice and policy in order to develop a conceptual framework. A targeted search was conducted in PubMed, Scopus, Web of Science and Google Scholar for peer-reviewed publications, with additional sources identified through reference chaining from key articles. Only English-language publications were considered. Search terms included a combination of dysphagia, TMFs, thickened foods, IDDSI, older adults, long-term care, care homes, nursing homes, malnutrition, nutrient intake, energy intake, protein intake, micronutrients, plate waste, sensory acceptability, mealtime assistance, nutrient density, food structure, rheology, moulding and three-dimensional food printing.

To complement academic literature, clinical, professional and practice-based documents were reviewed to contextualise how dysphagia nutrition is currently implemented in care homes. These included guidance and resources from the International Dysphagia Diet Standardisation Initiative (IDDSI), the National Health Service (NHS), the National Institute for Health and Care Excellence (NICE), Care Quality Commission (CQC), the British Dietetic Association (BDA), the British Geriatrics Society (BGS), the Malnutrition Task Force England, and the European Society for Clinical Nutrition and Metabolism. These sources informed the discussion of texture compliance, nutritional screening, menu planning, care home food service delivery and nutritional risk monitoring.

### Measurement gap: served food does not equal consumed food

Within residential care homes, nutritional governance is primarily structured around evidence of provision, including menu planning, recipe specifications, nutritional screening, and food-service documentation (15, 21). These approaches are essential to ascertain that meals are designed to meet established dietary requirements. However, it is also assumed that planned provision broadly reflects realised intake (22, 23).

For residents prescribed TMFs, intake is shaped by multiple physiological and behavioural constraints, including swallowing impairment, prolonged mealtimes, fatigue, dependence on feeding assistance, and reduced sensory appeal of foods. These factors limit the quality and pace of eating, which results in partial meal consumption (early cessation of meals). Under these conditions, menu-based estimates may not reflect realised intake (22, 23). Wright et al. (24) reported that older adults consuming TMFs experienced substantially greater deficits from estimated nutritional requirements than those consuming regular diets, with mean energy deficits of 2,549 kJ/day versus 357 kJ/day and protein deficits of 22 g/day versus 6 g/day, respectively. Similarly, a hospital-based plate-waste study reported mean plate waste of 47.5% among patients receiving TMFs, increasing to 65% for blended diets, indicating that substantial proportions of nutritionally planned meals remained uneaten (25).

Pureed meals may therefore be served in nutritionally planned portions yet only partially consumed because of fatigue, delayed feeding assistance, or reduced meal acceptance. Residents requiring feeding assistance additionally experience interruptions during meals, prolonged intervals between mouthfuls, or meals consumed at suboptimal temperatures, all of which can further compromise intake despite documented meal provision. Meal completion can also vary substantially between eating episodes, particularly among residents living with dementia, further weakening the relationship between planned provision and realised intake.

This divergence between planned nutritional provision and realised intake is consistently observed among individuals consuming TMFs. In aged-care residents consuming TMFs, only 35% met the estimated energy requirements, whereas almost half were at risk of malnutrition and more than one-third were already malnourished (16). These observations suggest that nutritional risk can persist despite therapeutic diets and nutritional supplementation. Indeed, meals satisfy documented standards while substantial proportions remain uneaten, creating a divergence between recorded provision and realised intake that is not routinely captured within quality indicators.

In practice, nutritional risk within care homes is commonly assessed using validated screening tools, such as the Malnutrition Universal Screening Tool (MUST), complemented by process indicators such as menu compliance, food-service documentation and verification of IDDSI-aligned textures and thickened fluids (26). Although these measures are valuable and widely implemented, they primarily identify established nutritional decline rather than emerging reduction in dietary intake (15). Weight loss and deterioration in screening scores often become apparent only after prolonged periods of insufficient intake, at which point vulnerability is already present (26). During this interval, routine monitoring provides limited visibility into intake adequacy at the level of individual meals.

Despite their direct relevance to realised intake, indicators such as meal completion, plate waste, interrupted feeding episodes, duration of assisted feeding, and food refusal are not consistently incorporated into routine quality assessment frameworks within long-term care (27). Consequently, nutritional deterioration can emerge gradually while remaining relatively invisible within systems primarily designed to monitor provision, documentation, and compliance. This creates a persistent served–consumed gap in which meals may satisfy planned nutritional targets while nutritional deterioration emerges through inadequately monitored reductions in intake.

### Production gap: texture modification can alter nutrient density and acceptability

Beyond limitations in nutritional measurement, food production within residential care homes introduces additional challenges in maintaining the nutritional and sensory quality of TMFs. Texture modification is essential for swallowing safety. When appropriately formulated, TMFs can meet established nutritional requirements. However, within institutional care homes, the preparation of IDDSI-compliant TMFs frequently occurs under operational pressures that prioritise safety, consistency, reproducibility, and service efficiency across large numbers of residents.

TMFs are prepared through processes such as blending, dilution, sieving and the incorporation of thickening agents to achieve target rheological properties. These approaches are necessary for swallowing safety but alter nutrient density and nutrient delivery per mouthful. For example, dilution with water, stock or gravy during blending, particularly in puree-based meals, can reduce energy and protein density per unit weight (28). Similarly, starch- or gum-based thickening agents, while effective in achieving desired viscosity and stability, contribute limited intrinsic protein, fibre or micronutrients relative to the ingredients they partially replace. This thickener displacement may reduce the overall nutrient density of TMFs (16).

Sieving and fine homogenisation may further remove structural components of foods, such as fibrous fractions and particulate food components that contribute to dietary fibre and micronutrient diversity, whereas repeated reheating and cook–chill production may further reduce heat-sensitive micronutrients, including vitamin C and folate (29). While each of these effects may be modest in isolation, their cumulative impact can reduce the nutritional value delivered per mouthful, particularly in meals that are already volume-constrained.

Micronutrient adequacy is an important but under-recognised component of the intake–adequacy gap in dysphagia nutrition. Older adults prescribed TMFs may consume smaller meal volumes because of swallowing difficulty, poor appetite and fatigue; prolonged mealtimes may further reduce intake by increasing fatigue and the likelihood of early meal termination. The risk may be greater in individuals with increased nutritional requirements or pre-existing deficiencies, such as those with osteoporosis or vitamin D deficiency, iron-deficiency anaemia, or pressure injuries requiring adequate zinc for tissue repair. Rodd et al. (7) highlighted the potential for TMFs to compromise micronutrient adequacy, particularly when intake volume is restricted. Similarly, Wu et al. (19) reported lower energy and protein intake, together with inadequate intake of several micronutrients, among individuals consuming TMFs and thickened fluids, indicating that the nutritional consequences of texture modification extend beyond macronutrient insufficiency (7, 16, 19).

Texture modification may further reduce micronutrient delivery through the physical and thermal processes used to achieve swallowing-safe consistencies. Dilution with water, stock or gravy may reduce micronutrient density per mouthful, while sieving and fine homogenisation may remove fibrous or particulate fractions that contribute to dietary fibre and micronutrients, including vitamin C and folate. Keller et al. (25) demonstrated that nutritional issues associated with TMFs include the risk that texture modification may reduce nutrient density, while William et al. (26) reported losses of heat-sensitive vitamins in cook–chill and cook/hot-hold food-service systems. These effects are particularly important in dysphagia care because residents with limited eating endurance may be unable to compensate for reduced micronutrient density by increasing meal volume. Micronutrient adequacy should therefore be assessed in relation to the nutrient density of the portion consumed. Further research should directly quantify labile micronutrients in prepared and consumed TMFs, as recipe-based estimates may not account for losses during processing, storage and reheating.

Furthermore, in addition to compositional changes, texture modification alters sensory properties that influence appetite, meal acceptance, and sustained intake. Conventional pureed foods are frequently presented as visually homogeneous portions in which the original structure of foods is lost, and individual food components become difficult to recognise. This loss of visual identity is clinically important because visual recognition, colour, shape and flavour cues contribute to anticipatory appetite responses before consumption begins (18–20). Reduced recognisability therefore weakens sensory engagement with meals, particularly among older adults already experiencing reduced appetite, cognitive impairment, fatigue and dependence on assisted feeding. Lam et al. (30) demonstrated that improving food recognisability and presentation increased meal acceptability and overall liking among individuals consuming TMFs. Similarly, Pu et al. (31) found that visually modified pureed foods were perceived as more appetising and acceptable than standard scoop-served purees among aged-care residents consuming TMFs, highlighting the contribution of food presentation and sensory appeal to meal acceptance. Chen et al. (32) demonstrated that food-shaping interventions that reshaped and visually enhanced TMFs consistently improved food recognisability, mealtime satisfaction and willingness to eat compared to conventionally presented pureed foods. These observations suggest that institutional food-service practices that prioritise texture consistency and production efficiency may inadvertently reduce nutrient density, visual appeal and meal acceptability.

### Care-system gap: translating nutritional standards into realised intake

Beyond limitations in measurement and food production, governance and procurement structures shape how dysphagia nutrition is operationalised within care homes. In the United Kingdom, care homes function within established regulatory and clinical frameworks that emphasise nutritional screening, dietetic input, therapeutic diet provision and alignment with population-level dietary guidance. However, these frameworks are less explicit on how planned nutritional provision should be translated into TMFs and assessed at the level of realised intake.

Guidance relating to nutrition and food service delivery within long-term care has traditionally focused on menu planning, regulatory compliance, food safety and standardisation of therapeutic diets. For example, the British Dietetic Association (BDA) Care Home Digest and the Dietitians of Canada Best Practices for Nutrition Food Service and Dining in long-term care homes emphasise menu auditing, nutrient analysis, allergen management and implementation of TMFs according to established swallowing safety standards (33, 34) Deasy et al. (35) reported poor adherence to British Association nutrition standards in Irish long-term care facilities, with some IDDSI-level foods providing only 1,323 kcal/day and 45 g protein/day relative to recommended targets of 1840–2,772 kcal/day and 79–92 protein/day for nutritionally vulnerable residents.

Food-service contracts, commissioning processes, and regulatory inspections, therefore, typically prioritise menu provisions, therapeutic diet availability, food safety and compliance with IDDSI texture standards because these indicators are easy to standardise, document and audit across services (Box 1) (26, 36). These priorities are appropriate for managing immediate and visible risks, including choking, aspiration and foodborne illness. In comparison, meal completion, nutrient density and actual consumption are more variable and difficult to assess within routine service delivery.

Box 1 **The swallowing safety–nutritional adequacy gap as a reinforcing systems dynamic**

Swallowing safety is readily auditable in routine practice, whereas nutritional adequacy is less directly visible. This imbalance may contribute to a reinforcing system in which TMFs remain safe but nutritionally fragile within care settings.

- **Differential auditability**

Texture and flow properties are defined by explicit, testable criteria and can be routinely verified at the point of service, whereas nutritional performance lacks an equivalent low-burden standard for verification at the point of intake.

- **Emphasis within quality assurance**

Quality assurance processes therefore tend to focus on texture compliance, labelling and documentation, while nutritional performance and intake-realised adequacy are less directly evaluated.

- **Alignment of procurement and service priorities**

Food-service specifications often reflect these priorities, emphasising texture compliance, food safety and operational efficiency, while nutritional characteristics such as density and variety may receive less explicit attention.

- **Effects of standardised production practices**

Common preparation approaches, including dilution, sieving and the use of thickening agents, support texture consistency but may also influence nutritional composition and sensory characteristics.

- **Downstream clinical response**

Nutritional concerns are often identified after measurable decline has occurred and may be addressed through supplementation rather than through modification of routine food provision.

- **Feedback through outcome-based indicators**

Nutritional adequacy is typically inferred from downstream indicators such as body weight, rather than from direct assessment of intake during routine care.

Together, these interacting processes may reinforce a system in which TMFs achieve reliable swallowing safety while variability in intake-realised nutritional exposure remains less visible.

This distinction is particularly relevant for TMFs because compliance with texture standards does not necessarily indicate adequate intake at the point of consumption. Individuals consuming TMFs consistently demonstrate lower energy and protein intake, increased plate waste, prolonged mealtimes and reduced meal satisfaction despite TMFs being designed to meet nutritional targets (16, 17, 24). Nevertheless, indicators relating to realised intake, including meal completion, interrupted feeding episodes, plate waste and adequacy of nutrient delivery per mouthful, are not routinely incorporated into procurement specifications and regulatory quality metrics within many care homes.

Within institutional food-service, factors such as production efficiency, consistency, shelf stability, portion control and reheating performance are prioritised because they facilitate reproducible and auditable service delivery across large resident populations. A key example of this is commercially prepared TMFs, which are designed to deliver reliable rheological properties and facilitate texture compliance; however, depending on formulation, many products rely heavily on dilution or starch- or gum-based thickening agents, resulting in limited protein, fibre and micronutrients compared to whole foods.

Therefore, oral nutritional supplements (ONS) are sought after within dysphagia nutritional management and are widely recommended for individuals at risk of malnutrition within existing clinical guidance (37). ONS function as adjuncts to otherwise nutritionally adequate meals; in others, they may compensate for reduced intake associated with low nutrient density, poor meal acceptability, prolonged mealtimes and incomplete meal consumption.

### Intake-realised nutritional adequacy in dysphagia care

The preceding sections indicate that, in dysphagia care, nutritional adequacy cannot be inferred solely from menu design; it depends on whether nutritional requirements are efficiently translated into TMFs that are safe to swallow and consumed in sufficient quantity over repeated eating episodes. Existing dietary frameworks within the UK were developed primarily to define population-level nutritional requirements and guide menu planning; however, they were not designed specifically to address the reduced intake capacity, prolonged mealtimes, altered food structures and swallowing-related difficulties that characterise dysphagia care. Consequently, nutritional adequacy in dysphagia is shaped by three interrelated domains: realised intake, nutrient density and swallowing compatibility (Table 2).

**Table 2:** Structural origins of the intake–adequacy gap in dysphagia care.

System domain	Underlying constraint	Consequence for intake and nutrient delivery	Implication for nutritional adequacy
Measurement systems	Reliance on provision-based indicators and retrospective screening frameworks	Limited resolution of intake dynamics and early-stage dietary decline	Nutritional adequacy inferred from provision rather than realised exposure
Food production processes	Dilution, homogenisation and reliance on viscosity-modifying agents during TMF preparation	Reduced nutrient density per unit volume and altered compositional balance	Nutrient delivery constrained within limited intake capacity
Sensory and perceptual properties	Reduced recognisability, monotony and altered flavour release associated with texture modification	Lower acceptability, reduced engagement and increased plate waste	Intake-mediated reduction in nutritional exposure
Physiological determinants	Swallowing fatigue, impaired oral processing and limited eating endurance	Early cessation of meals and incomplete consumption	Inability to compensate for low intake through volume
Food structural organisation	Weak integration of nutrients within dilute or poorly structured matrices	Inefficient incorporation of nutrients into the swallowed bolus	Reduced efficiency of nutrient delivery during consumption
Governance and quality systems	Emphasis on auditable safety, compliance and provision metrics	Limited visibility of intake performance within routine monitoring systems	Misalignment between documented provision and nutritional outcomes

Realised intake represents the principal determinant of nutritional exposure among individuals consuming TMFs (16, 38). Evidence from long-term care and hospitals consistently demonstrates prolonged eating duration, increased plate waste, reduced meal satisfaction and low energy and protein intake among individuals prescribed TMFs compared with regular diets (24, 39), largely driven by swallowing fatigue, cognitive impairment, dependence on assistance and reduced sensory appeal (40, 41). Under these conditions, menu-based estimates of nutritional provision substantially overestimate intake at the point of consumption.

Comparative studies further indicate that individuals consuming TMFs often achieve lower energy and protein intakes than those consuming regular diets, even when menus appear nutritionally adequate (42, 43). This variation reflects not only reduced intake, but also compositional changes introduced during conventional TMF preparation, including dilution during blending and the use of viscosity-modifying agents that contribute limited intrinsic nutritional value (22, 44, 45). As a result, nutrient delivery per unit volume is reduced, particularly when only a proportion of each meal is consumed.

In addition, the functional food form and structure influence intake and nutritional delivery. Visual appearance, recognisability and sensory variation contribute to appetite stimulation and sustained consumption, particularly among older adults experiencing sensory decline or cognitive impairment (32, 41, 46). Conventional texture modification processes, such as blending and homogenisation, reduce recognisability and produce visually homogeneous TMFs that diminish appetite and increase plate waste (30, 38). Such observations suggest that the nutritional performance of TMFs is shaped not only by nutrient composition, but also by the extent to which food structure and sensory presentation sustain eating engagement throughout the meal.

These considerations are closely linked to swallowing safety as the IDDSI framework provides standardised terminology and testing methods so that the foods meet appropriate texture and flow properties for safe swallowing. In practice, however, nutritional adequacy and swallowing safety are often addressed through parallel processes, i.e., nutritional requirements are addressed through menu planning and nutrient analysis, whereas texture compliance is assessed during preparation and service delivery. At the menu-planning level, texture properties are verified at the point of service. Consequently, the interaction between nutrient delivery, food structure, swallowing compatibility and realised intake is not always explicitly integrated within routine nutritional assessment. Reframing dysphagia nutrition around intake-realised adequacy, rather than planned provision alone, might therefore provide a more clinically effective framework for evaluating the effectiveness of TMFs within long-term care and institutional care (Box 2).

Box 2 **Reframing nutritional adequacy in dysphagia care: from provision to realised intake**

Nutritional adequacy in dysphagia care is commonly inferred from planned provision and downstream clinical indicators. However, the intake–adequacy gap highlights the need to reinterpret adequacy as an intake-dependent and context-sensitive outcome.

- **Adequacy as an intake-dependent outcome**

Nutritional sufficiency is determined by what is consumed rather than what is provided, particularly in individuals with constrained eating capacity.

- **Nutrient density as a functional requirement**

When intake is limited, the concentration of nutrients within the consumed volume becomes a primary determinant of adequacy rather than total daily provision.

- **Food structure as a mediator of nutrient delivery**

The physical organisation of food influences bolus formation, sensory perception and eating behaviour, thereby shaping intake-realised nutritional exposure.

- **Acceptability as a determinant of intake**

Sensory characteristics, recognisability and eating experience directly influence willingness to eat and sustained consumption.

- **Integration of safety and adequacy**

Swallowing safety and nutritional adequacy represent interdependent design limits requiring alignment at the level of formulation and consumption rather than parallel evaluation.

### Operationalising the intake-adequacy gap in practice

Although the intake–adequacy gap is conceptually apparent, its routine assessment remains underdeveloped in dysphagia care. Nutritional governance in hospitals and long-term care homes is still largely organised around planned provision, menu compliance, availability of therapeutic diets and retrospective indicators of nutritional decline, such as weight loss and malnutrition screening scores (5, 47). These measures are important; however, they do not necessarily determine whether a TMF that has been planned, prepared and served is consumed in sufficient quantity to deliver its intended nutritional value. Consequently, nutritional adequacy may be inferred from menu composition or the availability of IDDSI-compliant meals, even when a substantial proportion of the served meal remains uneaten (19).

This limitation reflects the fact that swallowing safety and nutritional adequacy are often assessed through separate systems. Texture compliance can be verified at the point of service using IDDSI descriptors and testing methods, which are intended to confirm the flow or textural characteristics of foods and fluids at the time of testing (5). In contrast, realised nutritional intake is more difficult to monitor because it is influenced by meal completion, appetite, fatigue, feeding assistance, food temperature, sensory appeal, cognitive status and eating behaviour across repeated meals (19). The transition from nutritional provision to actual nutrient exposure therefore remains insufficiently visible within routine care. As a result, nutritional deterioration may only become apparent after downstream outcomes, such as weight loss, deterioration in nutritional screening scores or increased reliance on oral nutritional supplements, have already occurred (47).

Existing literature provides some important examples, such as how different components of this gap could be assessed, although these approaches have not yet been integrated into a routine dysphagia nutrition pathway. These studies do not yet constitute an integrated routine assessment pathway, but they provide methodological examples for operationalising the intake–adequacy gap in practice (Table 3). Wright et al. (24) quantified 24-h dietary intake in older hospital patients using weighed food intakes and food record charts. In their study, 30 patients prescribed TMFs were compared with 25 patients consuming a regular hospital diet. The results from this study revealed that individuals on TMFs had significantly lower energy intake than those consuming regular diets, with mean intakes of 3,877 versus 6,115 kJ/day, respectively. Protein intake was also significantly lower in the TMFs group, at 40 versus 60 g/day. Importantly, when intake was compared with individual estimated requirements, the TMFs group has significantly larger energy and protein deficits than the regular diet group. This study illustrates how the intake-adequacy gap could be quantified by comparing realised dietary intake with estimated nutritional requirements, rather than assuming that the nutritional value of planned or served diet reflects actual nutrient exposure. Similarly, Razalli et al. (25) used plate-waste assessment in hospitalised patients receiving TMFs and showed that a considerable proportion of served food remained uneaten, particularly among patients receiving blended diets. Such findings also indicate that plate waste could provide a direct estimate of the discrepancy between served provision and consumed nutrition. Likewise, Shimizu et al. (17) reported that TMFs were associated with poor appetite among older adults admitted to post-acute rehabilitation, which indicates that appetite assessment may help identify individuals at risk of reduced realised intake. Additionally, studies investigating moulded, shaped, visually enhanced pureed foods have reported improvements in appearance, recognisability, overall liking, acceptability and mealtime experience, indicating that sensory and visual properties may influence whether TMFs are likely to be consumed (31, 32).

**Table 3:** Operationalising the intake–adequacy gap in dysphagia care: from planned provision to realised intake.

Population	Assessment approach	Principal findings	Relevance to the intake– adequacy gap	Reference
Older hospital patients prescribed TMFs compared with patients receiving regular hospital diets	Weighted food intake and food record charts; comparison with individual estimated energy and protein requirements	Patients receiving TMFs have significantly lower energy protein intakes than those receiving regular diets, with larger deficits from estimated requirements	Demonstrates that realised intake can be compared with estimated requirements to quantify nutritional shortfall among TMF consumers	Wright et al. (24)
Hospitalised patients receiving TMFs	Plate-waste assessment	A significant proportion of served food remained uneaten, with particularly high waste among patients receiving blended diets	Demonstrates that plate waste provides a practical measure of the discrepancy between served nutritional provision and consumed nutrition	Razalli et al. (25)
Older adults admitted to a post-acute analysis of a rehabilitation hospital	Appetite assessment and analysis of its association with TMF consumption	TMF consumption was associated with poor appetite	Identifies appetite as a measurable behavioural determinant of reduced realised intake	Shimizu et al. (17)
Long-term care residents	Assessment of TMF use, mealtime duration and dysphagia risk	Mealtime duration and dysphagia related eating difficulty were relevant to intake performance	Supports the use of mealtime process indicators that include eating duration and mealtime difficulty to identify intake-related risk	Namasivayam-Macdonald et al. (22)
Aged care residents and individuals consuming TMFs	Dietary intake assessment, nutritional status evaluation, evaluation, meal-provision analysis and mealtime-satisfaction measures	TMF consumers showed reduced intake, nutritional vulnerability, mealtime challenges and lower mealtime satisfaction	Supports monitoring of realised intake, nutritional status, meal competition and mealtime experience as components of intake adequacy	Wu et al. (19)
Acute hospital patients receiving moulded pureed diets	Evaluation of taste, appearance, recognisability and overall liking.	Moulded pureed diets improved recognisability, appearance, taste and overall liking	Indicates that sensory and visual acceptability may serve as practical indicators of likely consumption	Lam et al. (30)
Residents in aged-care facilities receiving modified puree meals	Feasibility and acceptability assessment of visually modified puree meals	Visually modified puree meals were perceived as more appetising and acceptable than standard puree meals	Demonstrates that presentation and recognisability may influence meal acceptance and realised intake	Pu et al. (31)
Older adults in aged-care settings; systematic review of food-shaping techniques	Synthesis of evidence on food-shaping, mealtime experience, nutrition and quality of life.	Food shaping approaches, improved recognisability, mealtime experience and willingness to eat	Supports food shaping as a potential intervention to improve acceptability and intake-related outcomes	Chen et al. (32)

Despite this evidence, there is currently no widely implemented framework that integrates texture compliance, nutrient density, meal completion, acceptability and nutritional outcomes within a single routine assessment pathway. In practice, a meal may be nutritionally planned and IDDSI-compliant but still fail as a nutritional intervention if it is diluted, poorly accepted, only partially consumed, or discontinued due to fatigue and inadequate feeding assistance. Operationalising the intake-adequacy gap therefore requires a shift from asking whether an appropriate TMF was provided to asking whether sufficient nutrients were actually consumed.

In care homes, the intake–adequacy gap might be examined through a structured meal-level assessment pathway that links nutritional provision, observed consumption and responsive intervention. This would require moving beyond documentation of menu provision and assessing whether the nutrients intended in a TMF are actually delivered at the point of intake. This could be operationalised as follows: (1) Establish the nutritional value of served TMFs: The nutrient composition of commonly served TMFs should first be determined using standardised recipes, menu-analysis software, food-service nutrient calculations and, where feasible, laboratory-based compositional analysis. This would define the nutritional value of the served portion, including energy, protein and key micronutrients; (2) record actual consumption at mealtimes. Actual intake should then be recorded during routine mealtimes using simple observational measures such as percentage of meal completed, plate waste, food refusal, eating duration, interruptions during assisted feeding, need for prompting, resident acceptability and whether the meal was consumed at an appropriate temperature. These measures would help identify whether nutritional provision is being converted into realised intake or lost through incomplete consumption; (3) Estimate realised nutrient intake. Realised intake should be calculated by adjusting the nutrient composition of the served portion according to the proportion consumed, enabling energy, protein and micronutrient intake to be quantified; (4) Use monitoring data to trigger targeted intervention. Where repeated monitoring identifies poor intake, high plate waste, poor appetite, or low acceptability, the findings should trigger targeted intervention. These may include recipe formulations, improved nutrient density, reduced dilution, micronutrient fortification, including protein fortification, portion size adjustment, improved mealtime assistance, and sensory enhancement of TMFs. Where poor intake is partly related to low recognisability, monotony, and poor visual appeal, moulding, shaping, and other presentation strategies such as three-dimensional printing technology may be used to improve food identity and mealtime engagement; and (5) create a feedback pathway across care teams. For implementation, care staff could document meal completion and food refusal during routine mealtimes; dietitians could review residents with repeated low intake; catering teams could adjust formulation and presentation; and speech and language therapists could review the appropriateness of the prescribed IDDSI level when low intake appears related to swallowing difficulty and fatigue. In this way, assessment would not simply document poor intake but also create a practical feedback loop among care staff, dietitians, catering teams and dysphagia specialists. Accordingly, operationalising the intake–adequacy gap would move the concept beyond a purely descriptive construct, enabling its use as a measurable indicator of nutritional performance and a practical basis for targeted intervention in dysphagia care.

### Linking food structure to nutrient delivery in TMFs

**Figure 2:**
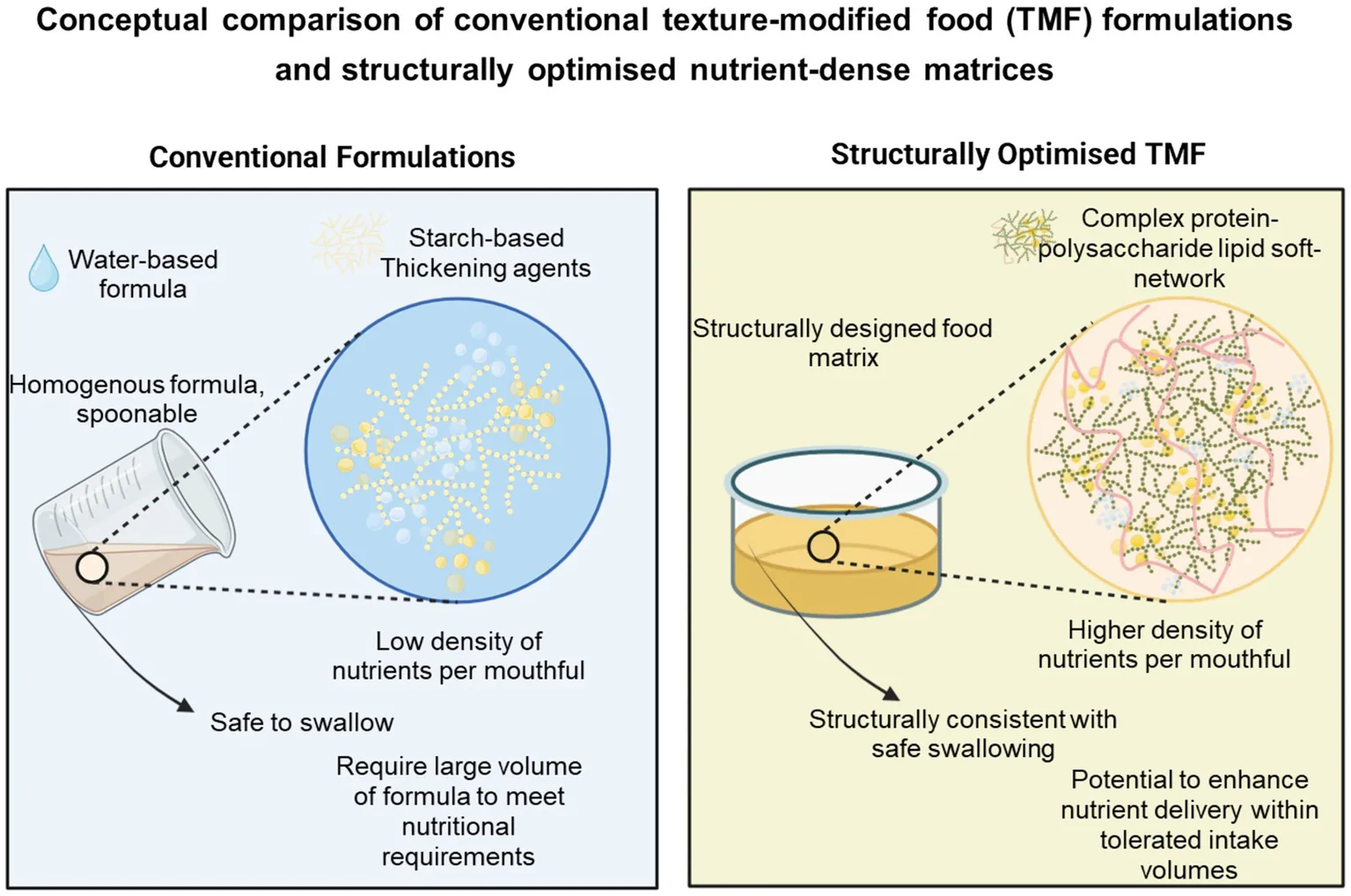
Structural determinants of nutrient delivery in texture-modified foods (TMFs). Conventional dilute TMFs are commonly stabilised using starch- or gum-based thickening systems and are associated with lower nutrient density per mouthful, whereas structurally designed TMFs incorporate integrated protein–polysaccharide–lipid matrices that simultaneously maintain swallowing-compatible rheological properties and enhance nutrient delivery under conditions of constrained intake.

The relevance of nutritional adequacy in dysphagia care therefore ultimately depends on how food structure mediates swallowing safety and nutrient delivery. In this context, TMFs might be more appropriately understood not simply as altered versions of conventional TMFs, but as structured foods in which composition and physical organisation together shape intake-realised nutrition (Figure 2). Such consideration is clinically important because many individuals with dysphagia are unable to compensate for reduced nutrient density through increased meal volume due to prolonged eating duration, fatigue and early satiety.

Emerging intervention evidence indicates that modification of TMF structure, enrichment and presentation may improve intake-related outcomes and acceptability. Ott et al. (48) evaluated a nutrition concept combining texture modification, enrichment and reshaping in 16 nursing-home residents with chewing and/or swallowing difficulties. Following implementation of the enriched and reshaped TMFs, daily energy intake increased by a median of 204.2 kcal, protein intake increased by 18.3 g, and body weight shifted from a median decrease of 0.5 kg during the usual-diet period to a median increase of 1.1 kg after the intervention. Although this proof-of-concept study requires confirmation in larger trials, it suggests that TMFs can be designed not only for swallowing safety, but also to improve nutrient delivery and nutritional status.

More recently, Peladic et al. (49) evaluated ready-made TMFs fortified with amino acids and vitamin D in 30 older out-of-hospital patients with IDDSI level 4 oropharyngeal dysphagia. Across 493 tastings, only 4% were interrupted due to minor adverse events, and 95% of dishes were consumed entirely. The intervention was also reported to reduce caregiver burden, particularly because food administration was less time-consuming. These findings suggest that fortified ready-made TMFs may support practical meal management and realised intake, although longer-term controlled studies are required. Evidence from studies of moulded, shaped and visually enhanced pureed foods further indicates that improvements in appearance, recognisability and acceptability may enhance the mealtime experience and support consumption (30–32).

In contrast, approaches based on protein–polysaccharide–lipid matrix design, emulsion structuring, encapsulation, and three-dimensional food printing remain grounded mainly in experimental food science evidence, particularly rheological, microstructural, and formulation studies. Although these approaches provide a scientifically plausible basis for developing nutrient-dense and swallowing-compatible TMFs, their clinical effectiveness remains to be established. Future studies should therefore evaluate these strategies in dysphagia populations using integrated outcomes, including IDDSI compliance, swallowing safety, nutrient density, meal completion, sensory acceptability, realised nutrient intake and nutritional status. The following discussion therefore considers food structure at three interrelated levels: composition design, microstructural organisation, and macrostructural behaviour, each of which may influence the capacity of TMFs to deliver nutrients safely and effectively under conditions of constrained intake.

#### Compositional design

At the compositional level, the nutritional performance of TMFs reflects the balance between ingredients that contribute to structure and those that contribute to nutrient provision. Conventional preparation approaches, including dilution and the use of viscosity-modifying agents, indeed facilitate texture compliance but reduce nutrient density per mouthful. From this perspective, nutrient density is determined not only by ingredient selection but also by the extent to which nutrients are incorporated within the food matrix and retained during oral processing.

This principle aligns with the design of multiphase food matrices in which proteins, lipids and polysaccharides interact to form structural networks that stabilise texture while simultaneously serving as carriers of energy, amino acids and micronutrients (50–52). In contrast, formulations dominated by free water and viscosity-modifying starches or gums tend to increase meal bulk without a proportional increase in intrinsic nutritional value (24, 39). Partial substitution of the aqueous phase with nutrient-bearing components, such as protein-rich bases and emulsified lipids, will increase energy and protein density while maintaining rheological properties compatible with safe swallowing (53). For example, legume-derived protein isolates, including lupin proteins, possess intrinsic gelation and emulsifying functionalities that facilitate the formation of cohesive dysphagia-compatible matrices while simultaneously contributing nutritionally relevant protein content, thereby reducing dependence on nutritionally dilute starch-and gum-based thickening agents (54).

In addition, strategies to increase micronutrient density, such as direct fortification with minerals including iron and zinc, may cause sensory challenges such as metallic or off-flavours that can reduce intake (55, 56). Therefore, the structural organisation of TMFs may also influence sensory perception, as the incorporation of nutrients within protein–lipid–polysaccharide matrices have the potential to modulate flavour release and mask undesirable tastes (57–59). Such interactions are important, given that reduced food recognisability, sensory monotony, and diminished flavour perception are associated with lower meal satisfaction, as discussed above.

Strategies to improve micronutrient delivery in TMFs may therefore combine nutrient density, swallowing safety and sensory acceptability. Food-based approaches may include the use of naturally nutrient-rich ingredients, such as dairy products, eggs, fish, legumes, pulses, green leafy vegetables, nuts or seeds, where these can be safely incorporated into IDDSI-compliant textures (60). Fortified dairy or fortified plant-based beverages may also be used as nutrient-bearing liquid phases during blending, replacing water or nutritionally dilute stocks to increase the delivery of calcium, vitamin D, vitamin B12, iron, zinc and other micronutrients within limited intake volumes (56, 61). Technological approaches may include micronutrient-fortified purees, protein-enriched matrices, emulsion-based systems for fat-soluble vitamins and encapsulation strategies to reduce undesirable metallic or bitter notes associated with minerals such as iron and zinc (50, 51). However, these approaches should be assessed not only by compositional analysis, but also by IDDSI compliance, sensory acceptability, meal completion and realised micronutrient intake.

#### Microstructural organisation

Beyond ingredient composition, the internal organisation of TMFs influence bolus cohesiveness, lubrication and nutrient retention during swallowing. Cohesive, structured matrices support water retention, maintain bolus integrity and provide more effective incorporation of nutritionally relevant components compared with dilute thickened TMFs (54, 62, 63). Such properties are important in dysphagia because bolus fragmentation and poor oral clearance impair swallowing efficiency and limit intake. Protein gelation or protein-stabilised emulsification, in which molecular crosslinking is formed during thermal or enzymatic aggregation, can generate soft structured matrices with improved water retention, bolus cohesiveness and texture properties compatible with dysphagia requirements. (54, 62, 63). These processes produce soft solids with measurable yield stress and improved water retention and structural cohesiveness, with dysphagia texture requirements (64). Within such matrices, dispersed lipid droplets can be used to further enhance energy density, lubrication and flavour perception through modulation of aroma release and oral coating behaviour (65–67). Importantly, these approaches allow nutritionally relevant components to become integrated into the physical architecture of the food rather than remaining suspended within dilute aqueous phases.

#### Macrostructural behaviour

At the macrostructure level, TMFs must balance deformability with structural integrity, allowing foods to be handled, transported and broken down during oral processing while maintaining cohesive bolus formation (68, 69). Moderate cohesiveness and controlled deformability support safe swallowing, whereas excessive homogenisation may disrupt structural features that contribute to bolus stability. TMFs can therefore be more appropriately conceptualised as soft structured solids, with rheological behaviour arising from organised food matrices rather than polymer-thickened water alone.

Structural design using shaping technologies such as moulding or three-dimensional (3D) food printing may further enhance recognisability and portion differentiation without compromising swallowing safety, as is evident from advanced research (41, 67–69). However, these approaches are most effective when applied to nutritionally robust matrices, as visual reconstruction alone cannot compensate for inadequate composition and structural integrity.

### Translating TMFs into realised nutritional intake

Swallowing safety, nutritional guidance, food preparation and individual eating behaviour therefore operate as interconnected elements that together determine nutritional exposure. Within care homes, these processes are distributed across professional and organisational boundaries: speech and language therapists define the limits of safe swallowing; dietitians interpret nutritional requirements; catering teams translate these requirements into meals; and residents ultimately determine intake through acceptance and consumption (70).

From this perspective, nutritional outcomes reflect the degree of alignment across these domains. Menu design establishes nutritional intent; food preparation shapes composition and structure; and service conditions influence how food is presented and consumed (71, 72). While existing systems effectively support swallowing safety and planned nutritional provision, the translation of these elements into intake-realised exposure is less explicitly captured. The combined assessment of swallowing safety and nutritional adequacy requires both dimensions to be examined within the same mealtime pathway. In clinical and institutional practice, this may involve confirming IDDSI compliance at the point of service, establishing the nutrient density of the served portion, recording the amount of food actually consumed, documenting food refusal, eating duration, acceptability and the level of feeding assistance required, and relating these observations to nutritional outcomes such as body weight, malnutrition screening scores and reliance on oral nutritional supplementation (73–75). Such an approach might help care teams determine whether inadequate intake is associated with an unsuitable texture level, insufficient nutrient density, poor acceptability, inadequate mealtime support or the combined effect of these factors. It may also provide a practical basis for coordinated intervention across speech and language therapy, dietetics, catering and care staff. In this way, TMFs would be evaluated not only as foods that meet swallowing-safety requirements, but as nutritional interventions whose effectiveness depends on the extent to which they are accepted and consumed.

Interpreting nutritional adequacy as a system-level outcome highlights how variability arises between what is planned, what is served and what is consumed. This perspective shifts nutritional adequacy from a property of diet design to an emergent outcome of a distributed care system, in which interactions between clinical decisions, food systems and individual behaviour determine realised intake. In this context, the intake–adequacy gap may be understood not as a failure of individual components, but as the consequence of incomplete alignment across interconnected clinical, nutritional and organisational processes. Nutritional adequacy is therefore not achieved through isolated decisions, but through the coordination of everyday practices across clinical, catering, and care environments. Addressing the intake–adequacy gap requires identifying where intended nutrition is lost between meal planning, preparation, serving and consumption. Because the causes of inadequate intake vary between individuals, intervention should be tailored to the specific barrier rather than applying a uniform approach. Strategies may include increasing nutrient density, minimising dilution during texture modification, improving sensory appeal and recognisability, monitoring plate waste and meal completion, and providing appropriate mealtime assistance. Reformulation may be required when nutrient density is inadequate; texture review when swallowing effort or fatigue limits intake; flavour or presentation modification when acceptability is poor; and additional feeding support when assistance is insufficient. The intake–adequacy gap may therefore serve as a practical framework for identifying individual causes of inadequate intake and guiding targeted intervention in dysphagia care. Similarly, food-service systems, procurement standards and formulation strategies may need to prioritise nutrient delivery per mouthful, sensory engagement and practical feasibility alongside swallowing safety and production efficiency. Such a shift would reposition TMFs not simply as compensatory therapeutic diets, but as integrated nutritional interventions designed to sustain adequate intake when eating capacity is constrained.

## Conclusion

The standardisation of dysphagia diets through the International Dysphagia Diet Standardisation Initiative (IDDSI) has strengthened the safety, consistency and communication of swallowing management, illustrating how complex clinical risks can be translated into routine practice. Nutritional care in long-term care, hospital and community settings is also informed by established dietary frameworks; however, these standards do not fully converge at the point of consumption, where nutritional adequacy is ultimately determined by what is consumed rather than what is planned or served. Dysphagia nutrition is therefore less constrained by the absence of standards than by the incomplete translation of nutritional intent into food formulation, meal delivery and realised intake. Recognising nutritional adequacy as an intake-dependent outcome emphasises the importance of food composition, nutrient density, food structure, sensory acceptability and mealtime support in shaping consumption. Accordingly, outcomes for individuals with dysphagia will depend on the extent to which TMFs can support both swallowing safety and sufficient nutritional intake under conditions of constrained consumption. Addressing the intake–adequacy gap will require integrated assessment of IDDSI compliance, nutrient delivery per mouthful, actual meal consumption, food acceptability and nutritional outcomes. Future work should therefore develop and apply methods that directly capture intake-realised nutritional exposure, including meal completion, nutrient density, eating persistence, plate waste and patient-centred acceptability. Such approaches would strengthen the alignment between nutritional provision and nutritional outcomes in dysphagia care.
